# Results From a Multimethod Exploratory Scale Development Process to Measure Authoritarian Provider Attitudes in Democratic Republic of Congo and Togo

**DOI:** 10.9745/GHSP-D-22-00421

**Published:** 2023-11-30

**Authors:** Martha Silva, Kathryn Spielman, Leanne Dougherty, Sethson Kassegne, Amanda Kalamar

**Affiliations:** a Department of International Health and Sustainable Development, School of Public Health and Tropical Medicine, Tulane University, New Orleans, LA, USA.; b Population Council, Washington, DC, USA.; c Department of International Health, Johns Hopkins Bloomberg School of Public Health, Baltimore, MD, USA.; d Cera Group, Lomé, Togo.

## Abstract

**Background:**

Health care providers' actions can significantly influence clients' experiences of care, adherence to recommendations, and likelihood of re-engaging with health services. There are currently no validated scales that measure provider attitudes that could affect service delivery in multiple health areas.

**Methods:**

We developed provider attitude measures in 3 phases. In phase 1 (2019), survey items were developed based on literature reviews, and quantitative items were tested through a health facility survey conducted in the Democratic Republic of the Congo (DRC). Health care providers (N=1,143) completed a 23-question survey focused on 3 subdomains: provider perceptions of clients, provider roles, and gender roles. In phase 2 (2021), cognitive interviews were administered to 17 health care providers in DRC to assess and improve respondents' understanding and interpretation of questionnaire items and response options. In phase 3 (2021), 52 family planning providers were sampled from urban health facilities in Togo to retest and validate the improved measures.

**Results:**

Phase 1 showed the provider attitude items had low scale reliability, and 8 survey items had low variability. In phase 2, results from the cognitive interviews of the 21 items retained from phase 1 found 16 questions were not well understood or had low response variability and thus modified, and 4 survey items were added to test different iterations of specific survey items. In phase 3, exploratory factor analysis resulted in 1 provider attitude scale of 14 items reflecting authoritarian attitudes related to the 3 initial subdomains.

**Conclusion:**

This research highlights the importance of iteration and testing during scale development, implementable even across geographic locations. Provider behavior change programming should consider how authoritarian provider attitudes pertaining to professional roles, their clients, and gender norms may interact and influence the quality of health services provided.

## Abstract (precis)

Provider attitudes are a recognized driver of provider behaviors that may influence client behaviors & outcomes–the authors present a scale development & validation process resulting in survey items measuring authoritarian provider attitudes.

## Teaser

**Key Findings:**

- There is growing interest in understanding what drives providers' behaviors and attitudes toward clients and how these behaviors and attitudes are important to client health outcomes.
- Iteration and testing with target populations were key elements of the scale development process, starting with testing 23 items and retaining 14 items in the scale that measures authoritarian provider attitudes.
- Measuring provider attitudes using validated scales such as the scale presented in this article can identify areas for programmatic improvement by helping stakeholders understand drivers of provider behavior.

**Key Implications:**

- Public health practitioners working to measure and improve provider behavior may consider expanding their scope to broader attitudes that may influence behavioral drivers relevant to multiple health areas.
- Designers of provider behavior change programs should consider how authoritarian provider attitudes interact and affect the quality of health services.

## INTRODUCTION

Health care providers (HCPs) include a wide array of individuals—both formally trained through an accredited program and otherwise—that provide services, products, or information related to health and are thus in direct contact with clients and in a position to influence their health-related behaviors.^1^ HCP behaviors—a range of actions, from adherence to clinical protocols to client-provider interactions, such as listening to and responding to client questions, that characterize high-quality services—can significantly influence clients' experiences of the service, influence their likelihood to adhere to treatment or recommendations, and potentially alter clients' likelihood to re-engage with health services for improved health outcomes.^2^^–^^4^ For this reason, there is growing interest in understanding what drives providers' behaviors and which of these behaviors are key to clients' health outcomes.^5^

In addition to conditions related to structural or process factors, such as those identified by Donabedian's quality of care model,^6^ it is recognized that the behavior of HCPs is influenced by factors beyond their clinical training or knowledge of guidelines, including their experiences and manifestations of power, norms, attitudes, biases, expectations, and motivations.^4^^,^^5^^,^^7^^,^^8^ Social and behavior change programs can address the wide array of factors influencing provider behaviors. Factors such as providers' core beliefs, local values and norms, empathy for clients, and perceptions of their role are referred to as predisposing factors, which can shape attitudes toward clients or services and impact behaviors.^9^

Health programs routinely consider behavioral constructs, such as individual attitudes, norms, and self-efficacy, when identifying strategies to improve health behaviors.^10^ These behavioral constructs form the building blocks of established behavioral theory and are grounded in evidence. To measure these constructs, researchers formulate individual questions that, when measured collectively, provide an empirical basis for the construct. While empirically grounded measures have been developed to assess constructs related to an individual's health-seeking behavior, there is limited evidence on measures relevant for HCP behaviors.

Among the limited studies that consider behavioral constructs of HCPs, the majority use single-item questions that measure attitudes toward clients or attitudes toward a service, such as abortion^11^ or vasectomy,^12^ or a product, such as intrauterine devices^13^ or emergency contraceptive pills.^14^ Scales developed for other audiences, such as the Homophobia Scale, have also been adapted for use with HCPs, specifically in the context of HIV/AIDS.^15^ Measurement of HCP attitudes toward clients seeking contraception is often considered through the lens of medical eligibility criteria for contraception and assesses a provider's potential biases in providing contraception to women who were unmarried, nulliparous, young, or did not provide their partner's consent.^16^^,^^17^ While these studies offer insights into provider behaviors, the measures are not based on empirically grounded scales to assess the underlying behavioral construct. In fact, few studies have developed and tested empirically grounded scales to assess behavioral constructs influencing HCP behavior. Among the limited studies available, the focus has been on understanding HCP attitudes toward people with disabilities,^18^ attitudes toward the provision of abortion services,^19^ and HCP job satisfaction.^20^ We identified only 1 study that considered HCP self-efficacy for service provision, attitudes toward clients, and job satisfaction using tested scales.^21^

Few studies have developed and tested empirically grounded scales to assess behavioral constructs influencing HCP behavior.

We describe the process of developing reliable and valid measures for provider attitudes that may drive HCP behavior across multiple health areas, with provider attitudes being a single predisposing factor among many influencing behavior. We explore 3 provider attitude domains—provider perceptions of clients, attitudes about providers' roles, and attitudes about gender roles—and ultimately combine them into an overarching domain of authoritarian attitudes.

## METHODS

Provider attitude measures were developed in 3 phases by the Breakthrough RESEARCH project funded by the U.S. Agency for International Development. In phase 1, survey items to measure provider attitudes related to the 3 behavioral domains of interest were developed, quantitatively tested, and assessed for reliability and variability. In phase 2, cognitive interviewing was used as a technique to improve the survey items. Finally, in phase 3, the improved measures were quantitatively retested and analyzed through exploratory factor analysis.

### Phase 1: Survey Item Development and Testing

We followed a deductive process for domain and item generation in phase 1.^22^ We conducted a literature review to identify existing measures and constructs capturing behavioral barriers that can influence provider behaviors across contexts. A prior review conducted by Breakthrough RESEARCH had identified provider attitudes toward clients and attitudes toward professional roles as being susceptible to (1) fundamental attribution error—the tendency to explain others' behavior by intrinsic personality traits rather than by contextual features; and (2) mental schemas—whereby providers form representations or unconscious mental structures of how the world around them works and their relationship to it that are shaped by prior experiences and guide decisions and actions.^8^ Thus, the domains of attitudes toward clients and attitudes about professional roles were deemed highly likely to lead to HCP behaviors that might affect service delivery.

As we did not find validated scales for these 2 domains, survey items were developed de novo. In addition to these 2 domains, a third domain related to gender was included because of its importance as a social determinant of health.^23^ Survey items related to gender roles were adapted from the Gender Equitable Men scale.^24^^,^^25^ The domains of attitudes toward clients, attitudes toward professional roles, and attitudes toward gender roles were further validated as important elements of HCPs' behavioral ecosystem. Due to funding and operational constraints, the deductive item generation approach was not combined with an inductive approach based on qualitative interviews, as is best practice.^22^ We developed 23 items to measure provider attitudes across the 3 behavioral domains hypothesized to influence provider behavior: provider perceptions of clients, provider roles, and gender roles (Supplement Table S1).^26^ All items were developed in English and translated into French.

We tested the survey items in an HCP survey conducted in 2019 by the Data for Impact project, funded by the U.S. Agency for International Development.^27^ The HCP survey was conducted in 6 Democratic Republic of Congo (DRC) provinces (Sud Kivu, Tanganyika, Kasai Oriental, Sankuru, Haut Katanga, and Lualaba), where a total of 1,143 primary HCPs responded to a survey in French. Response variability across the 5-point response options was assessed for each item, as well as scale consistency using Cronbach's alpha. From this, 21 items were retained for further testing, and 2 items were dropped based on low item response variability.

### Phase 2: Survey Item Improvement

In phase 2, we conducted cognitive interviews with HCPs in French in DRC in 2021 as part of a larger mixed methods study led by the Breakthrough ACTION project. The purpose of the cognitive interviews was to assess and improve respondents' understanding and interpretation of questionnaire items and response options and to test and strengthen content and construct validity of the 21 provider attitude survey items. Cognitive interviews assess respondents' understanding and interpretation of questionnaire items and response options and are commonly used to develop, improve, and validate items for scale construction.^28^ Cognitive interviewing can offer insights into the processes that respondents use to answer questions that might ultimately bias the data. A better understanding of this process helps to illuminate potential response error in quantitative measures. Cognitive interviewing uses a series of qualitative probes to assess comprehension, recall, and judgment.^29^

A sample of 20 providers of any cadre was purposively selected from facilities with a median of more than 30 first antenatal care visits per month. Three interviews were omitted because interviewers conducted on-the-spot translation of the interview guide from French to Kiswahili, which may have introduced interviewer error.

The study team conducted thematic analysis on the 17 interviews to assess content and construct validity. Codes were developed for the type of misperception or misinterpretation present in respondents' explanations and responses to the survey items. This analysis was also complemented by triangulating quantitative data from the study fielded by the Data for Impact project in 2019. We assessed the response variability of each survey item and defined items as having (1) low variability in responses when 90% or more of responses fell into collapsed categories of agree or disagree; (2) acceptable variability when 70%–90% of responses fell into a collapsed category of agree/disagree; and (3) high variability when 50%–69% of responses fell into these categories. Variability in this context is necessary for explanatory power; thus, items with low variability were revisited during phase 2 analysis for reframing.

Based on this analysis, we identified potential updates to some of the survey items to enhance their content and construct validity, including different iterations of wording for select survey items, resulting in an addition of 4 items for a total of 25 items retained for testing in phase 3.

### Phase 3: Survey Item Retesting

#### Study Context

In phase 3, 25 survey items (including 21 of the initial items, after 2 were dropped, and the additional 4 wording iterations of select items resulting from phase 2) were retested as part of a family planning (FP) quality of care evaluation conducted in Togo in 2021, led by Breakthrough RESEARCH. In the context of this study, 52 FP providers who were primarily nurses or midwives providing FP services were sampled from urban health facilities in 6 districts (Golfe, Kozah, Ave, Zio, Tchaoudjo, and Agonyive). Trained interviewers administered a structured interview in French that included provider education and experience, trainings (capacity-building), working conditions, and experience with supervision as determinants of provider motivation, in addition to the updated provider attitude survey items.

#### Scale Testing

The analysis of the survey items in phase 3 began with recoding all positively/equitably framed items in the opposite direction so that higher responses reflected more inequitable attitudes. With the recoded items, we used exploratory factor analysis with iterated principal factor and parallel analysis with a scree plot generated to determine the number of latent factors. Items were reduced by retaining items with factor loadings greater than 0.35 on the factors identified in parallel analysis. We next assessed reliability of the resulting items and dropped any items with item-test and item-rest correlation below 0.35 that would result in greater overall reliability if dropped. A mean score was generated on the final items.

### Ethical Approval

Ethical approval for the Data for Impact study in which phase 1 survey items were tested was obtained from the ethics committees of Tulane University School of Public Health (2019-1279) in the United States and the Kinshasa School of Public Health (ESP/CE/216/2019) in DRC.

The study led by Breakthrough ACTION, in which phase 2 interviews were embedded, received ethical approval from the Johns Hopkins Bloomberg School of Public Health Institutional Review Board (Study #14438) in the United States and the Kinshasa School of Public Health ethics committee (ESP/CE/199/2020) in DRC.

The study led by Breakthrough RESEARCH, in which the provider attitude measures were embedded for retesting (phase 3), received ethical approval from the Population Council (Study #985) Institutional Review Board in the United States and the Ministere de la Sante et de l'Hygiene Publique et de l'Access Universel aux Soins du Togo.

## RESULTS

### Phase 1: Initial Survey Item Testing

Table 1 provides a description of the study participants included in each phase of the scale development process. In phase 1 and phase 2, the HCPs sampled were predominantly male (approximately 60% male and approximately 40% female). In phase 3, the HCPs were predominantly female (92%). In phase 1 and phase 2, the majority of HCPs were nurses, but in phase 3, the majority of HCPs were auxiliary birth assistants or midwives. The mean age of HCPs was 42 years in phase 1 and 37 years in phase 2. The age of HCPs was not collected in phase 3. The mean number of years as a provider was 12 in phase 2 and 10 in phase 3 (this item was not collected in phase 1). The mean number of years as a provider in the facility was 7 in phase 1, 5.5 in phase 2, and nearly 5 years in phase 3.

**TABLE 1.** Sample Description in Scale Development Process to Measure Authoritarian Provider Attitudes in Democratic Republic of Congo and Togo

	Phase 1, No. (%) (N=1,143)	Phase 2, No. (%) (N=17)	Phase 3, No. (%) (N=52)
Sex			
Female	458 (40.1)	5 (29.4)	48 (92.3)
Male	684 (59.9)	11 (64.7)	4 (7.7)
No response	-	1 (5.9)	-
Cadre			
Nurse	829 (72.6)	15 (88.2)	3 (5.8)
Doctor	138 (12.1)	1 (5.9)	-
Trained birth attendant	129 (11.3)	-	-
Laboratory technician	-	1 (5.9)	-
Other training	33 (2.9)	-	8 (15.4)
Auxiliary birth assistant	-	-	19 (36.5)
Midwife	-	-	22 (42.3)
Mean age, years (SD)	42.1 (10.7)	37.3 (7.3)	-
Mean no. of years as provider (SD)	-	12.0 (5.1)	10.3 (7.4)
Mean no. of years as provider in facility (SD)	7.0 (7.2)	5.5 (4.2)	4.9 (5.8)

Abbreviation: SD, standard deviation.

The provider attitude items initially developed did not work together as a scale as intended (Cronbach's α<0.60 for each domain: provider perceptions α=0.4925, provider roles α=0.4043, gender norms α=0.5766). Eight of the 23 survey items were found to have low response variability (items 3, 4, 9, 13, 15, 19, 20, and 22 [Table 2]). Two survey items were dropped after phase 1 analysis and before phase 2: item 3, which had the lowest response variability (“I consider my patients to be worthy of respect no matter how poor or low status they are”), and item 17 (“It is the man who takes the initiative to have sex with his wife”). Item 17 was dropped to be consistent with our objective of measuring provider attitudes that are not specific to a particular health area, as this item may reflect attitudes too specific to sexual and reproductive health.

**TABLE 2.** Evolution of Survey Items to Measure Authoritarian Provider Attitudes in Democratic Republic of Congo and Togo

Item No.	Phase 1	Phase 2	Problems Found in Phase 2	Phase 3 Fielded Version
Providers' attitudes associated with their perceptions of clients				
1	Patients I care for are not educated enough to make good health decisions for themselves.	No change	Question requires more subjectivity to reflect an attitude (i.e., capability vs education).	Patients I care for are not capable of making good health decisions for themselves.
2	Patients I care for are not grateful for the efforts I make when I care for them.	Patients I care for are not grateful for the efforts I make when I care for them.	Question requires more subjectivity to reflect an attitude (i.e., should).	Patients I care for should appreciate my efforts when I care for them.
3	I consider my patients to be worthy of respect no matter how poor or low status they are (low response variability).	Dropped	Dropped	Dropped
4	Patients often treat me without respect, so it's hard to treat them with respect (low response variability and double-barrel statement).	It's hard to treat patients with respect if they don't treat me with respect.	Low variability in phase 1 quantitative test. Question requires more subjectivity to reflect an attitude (i.e., should) and test variation focused on client's behavior.	a. One should treat patients with respect even if they don't treat me with respect. b. Patients must always respect providers, regardless of the quality of care they receive.
5	Patients I care for make bad decisions regarding their health no matter what I tell them.	No change	Question wording requires an element of judgement to reflect an attitude.	My patients don't listen to my advice no matter what I tell them.
6	My patients will work hard to improve their health when they are given the proper information.	My patients will put a lot of effort into improving their health if they are given the right information.	No change needed.	My patients will put a lot of effort into improving their health if they are given the right information.
Providers' attitudes about their professional role				
7	My role is to provide clinical care, not to teach patients about how to take care of themselves.	A provider's role is to provide clinical care, not to teach patients about how to take care of themselves.	More specificity.	A provider's role is to diagnose patients and provide clinical care, not to teach patients how to improve their health and prevent disease.
8	I do not spend a lot of thought about what patients may think about their experience at the clinic as I have other things to worry about.	No change	Frame in terms of responsibility, add more precision.	My responsibility is to diagnose and ensure appropriate treatment, not ensure they have a pleasant experience at the clinic.
9	An important part of my job is to communicate with patients to make sure they understand their care (low response variability).	An important part of success at my job is to communicate with patients to make sure they understand their care.	Low variability but no clear alternative based on CI. Testing out variations that test bidirectional communication and holistic view of HCP role.	a. I have the responsibility to ensure that patients have a say in their care. b. It is important to listen to patients to ensure they understand their care. c. It is my role to think about other elements of health care services, not just diagnosis and treatment.
10	I try hard to think about all of the patients' health care needs not just solving their immediate problem.	No change	Inconclusive CI result. Retest item, plus test wording framed as providers' responsibility.	a. I make an effort to think about all my patient´s needs regarding medical care, not just the immediate health problem. b. My role as a provider is to resolve my patients' immediate medical problems, and nothing else.
11	I was trained to provide clinical care, being respectful to every patient is not my job.	Providing respectful care is less important than providing effective clinical care.	Reverse statement for a more pronounced moral hierarchy.	It's more important to provide effective clinical care than it is to provide respectful care.
12	When medicine is given, it is important that I explain well what it does for the patient and how it helps them.	No change	Low variability but no indication of change needed due to CI, possibly wrong construct to measure.	When medications are given, it is important that I explain well to patients how they work and how it will benefit them.
13	I think it is important to spend enough time with each patient, even if I have other job demands (low response variability).	No change	Low variability, more specificity about time spent.	It is important to spend time putting patients at ease, even on a busy day.
14	My job is to diagnose and treat parents not to be a health educator.	My job is to diagnose and treat patients not to be a health educator for each patient.	No change needed.	My job is to diagnose and treat patients, not to be a health educator for each patient.
15	Engaging patients in discussions leads to better health outcomes than just telling them what is best for them (low response variability).	No change	Low variability but no indication of change needed due to CI, possibly wrong construct to measure.	Engaging patients in discussions leads to better health outcomes than just telling them what is best for them.
Providers' attitudes on gender norms				
16	A man should have the final word about decisions in his home.	A man should have the final say on decisions made in his home.	No change needed	A man should have the final say on decisions made in his home.
17	It is the man who takes the initiative to have sex with his wife.	Dropped	Dropped	Dropped
18	A woman's most important role is to take care of her home and cook for her family.	A woman's most important role is to take care of her home and cook for her family.	Align with GEM wording	A woman's most important role is to take care of her home and her family.
19	If a woman has a good idea, her husband should listen even if he disagrees (low response variability).	No change	Low variability	A woman must obey her husband in everything
20	Men and women should decide together about how many children to have (low response variability).	No change	Low variability Change to another GEM statement	It is important for men to be present in their children's life, even if he isn't with their mother anymore.
21	A man is expected to discipline his women.	No change	Change to another GEM statement	Sometimes a man must put his woman in her place.
22	Men should help take care of the children in the household (low response variability).	No change	No variability More specificity	A woman is the only one responsible for changing diapers, bathing and feeding her children.
23	There is never a good reason for a man to beat his wife.	No change	No change needed	There is never a good reason for a man to beat his wife.

Abbreviations: CI, confidence interval; GEM, Gender Equitable Men scale; HCP, health care provider.

### Phase 2: Improving Content and Construct Validity

In phase 2, 21 items were tested for content and construct validity. Before conducting the cognitive interviews, the study team reworded 1 item (item 4) to eliminate the double-barreled wording. Results from the cognitive interviews found that 5 of the 21 survey items had good content and construct validity and required no proposed revisions. The analysis identified 12 questions where there were instances of misinterpretation of the intent of the question, and modifications to the questions were made to address these issues. In addition to these cognitive interview findings requiring survey item changes, all 7 remaining questions with low variability in the phase 1 quantitative test were also modified based on qualitative information gathered during the cognitive interviews that suggested how to elicit greater response variability. Table 2 shows the evolution of survey items from phase 1 to phase 3 based on these phase 2 findings.

### Phase 3

#### Factor Analysis

We identified 2 latent factors from the initial exploratory factor analysis and scree plot with parallel analysis. Dropping items with factor loadings less than 0.35 on either factor 1 or 2 resulted in 16 total items, with 14 items loading onto factor 1 and 4 items loading onto factor 2; 2 items overlapped across factors. When assessing overall reliability and individual item alphas, the 2 unique items in factor 2 had item-test and item-test correlation below 0.35 and resulted in greater overall reliability if dropped. Thus, 14 items were retained, all loading onto factor 1, with each item's factor loading greater than 0.35 (Table 3; French version of the final survey instrument is available in Supplement Table S2). Reliability (assessed using Cronbach's alpha) of the final 14 items was α=0.8323. Upon review of the items retained onto factor 1, we identified authoritarian attitudes as the overarching domain based on the observed elements of dominance, inflexibility, and deprioritization of others' autonomy in the retained items. While this has some overlap with the original 3 conceptualized themes, we note that since the 3 themes did not emerge in the data in phases 1 and 3, it is likely that this single factor is capturing important aspects of provider attitudes within the single concept of authoritarian attitudes. The average mean score, from 1 to 5, for the 14-item provider attitude scale was 1.99 (SD: 0.50; range=1–3.43), meaning that, on average, the attitudes of these providers tended to be less authoritarian. We did not separate the positively and negatively worded items and saw that they were unidimensional.

**TABLE 3.** Scale Item Means With Item Test Correlation Developed to Measure Authoritarian Provider Attitudes in Democratic Republic of Congo and Togo

Item	Factor Loadings	Mean (SD)
1. Patients I care for are not capable of making good health decisions for themselves.	0.4702	2.06 (0.92)
2. Patients I care for should appreciate my efforts when I care for them.	0.5076	3.10 (1.29)
4a. One should treat patients with respect even if they don't treat me with respect.	0.4392	1.50 (0.61)
4b. Patients must always respect providers, regardless of the quality of care they receive.	0.6631	3.10 (1.32)
6. My patients will put a lot of effort into improving their health if they are given the right information.	0.3709	1.67 (0.76)
7. A provider's role is to diagnose patients and provide clinical care, not to teach patients how to improve their health and prevent disease.	0.5497	1.75 (0.84)
9a. I have the responsibility to ensure that patients have a say in their care.	0.7605	1.56 (0.61)
9b. It is important to listen to patients to ensure they understand their care.	0.4487	1.44 (0.50)
10b. My role as a provider is to resolve my patients' immediate medical problems, and nothing else.	0.4661	2.06 (0.94)
12. When medications are given, it is important that I explain well to patients how they work and how it will benefit them.	0.4305	1.46 (0.50)
14. My job is to diagnose and treat patients, not to be a health educator for each patient.	0.7185	1.88 (0.92)
16. A man should have the final say on decisions made in his home.	0.6195	2.46 (1.34)
19. A woman must obey her husband in everything.	0.6149	2.46 (1.34)
20. It is important for men to be present in their children's life, even if he isn't with their mother anymore.	0.4334	1.40 (0.50)

Abbreviation: SD, standard deviation.

We identified authoritarian attitudes as the overarching domain based on the observed elements of dominance, inflexibility, and deprioritization of others' autonomy.

#### Authoritarian Attitudes Scale

The resulting 14-item scale loaded onto 1 factor and comprised items from all 3 initial domains of provider attitudes: client perceptions, provider roles, and gender norms. We reviewed the items dropped and retained to find a common theme that this single latent construct was likely measuring. The theme that emerged from this qualitative review was that the retained items reflected authoritarian attitudes with respect to the 3 domains.

## DISCUSSION

We have described a survey item testing process, beginning with 23 items tested in DRC, followed by qualitative cognitive interviewing to improve scale validity and reliability, and ending with 14 survey items retained as a scale that measures authoritarian provider attitudes that include elements of 3 domains: attitudes toward clients, attitudes toward their professional role, and attitudes toward gender norms. While confirmatory factor analysis is still needed, this exploratory factor analysis is the first of its kind for health-topic-agnostic HCP attitudes. We hypothesize that the attitudes we measured will be related to attitudes about FP and other types of health care, as well as behavioral outcomes, such as FP uptake.

Authoritarian attitudes have been measured in the past using the Authoritarianism Scale, a revised version of the F scale,^30^ including 4 factors: dominance/leadership, achievement/motivation, interpersonal conflict, and verbal hostility.^31^^,^^32^ Despite the existence of these validated measures of authoritarian attitudes, there have been relatively few efforts to include them in the field of public health as explanatory variables to study or address attitudes as drivers of provider behavior concerning service delivery or quality of care. Most studies of authoritarian attitudes lie in the field of education and psychology and center on parenting,^33^^,^^34^ mental health, and addiction.^35^^–^^37^

There have been relatively few efforts to include validated measures of authoritarian attitudes in public health as explanatory variables to study or address attitudes as drivers of provider behavior concerning service delivery or quality of care.

Recent strides have been made in testing multipronged strategies to change HCP attitudes and beliefs to be less biased against youth and adolescents seeking FP services.^17^ However, a recent rapid review of measurement of provider behavior and behavioral determinants found that most provider behavior change interventions rely heavily on training as an intervention for behavior change,^38^ even though mental schemas that manifest in attitudes may be less easily changed by training alone.^8^ Furthermore, trainings and values clarification exercises tend to focus on attitudes specific to health areas, such as attitudes toward FP methods or client restrictions to FP services. We hypothesize that provider attitudes that are further removed from a specific health area may be influencing other more health-specific beliefs. Public health practitioners working to measure and improve provider behavior may consider expanding their scope to broader attitudes, such as those explored in this article, which may influence FP-specific and other behavioral drivers. Furthermore, future implementation research should (1) examine how power among community subgroups and facility providers (i.e., different cadres) intersects with and varies by gender and (2) assess the influence that provider behavior change programs have on shifting power dynamics between HCPs and communities.^7^ Additionally, further testing is needed with community health workers or other community-level providers to adapt the scale to non-facility-based providers. Research shows that community health workers are considered similar to their clientele in shared demographics, experience, and location and different from facility-based HCPs, as they have more limited responsibilities and less training in duration and intensity.^39^^,^^40^ The difference in roles and responsibilities would need to be explored for in future testing of this scale.

### Limitations

This scale development process involving 3 different studies has several limitations. First, although the identification of subdimensions was based on a previous literature review conducted by Breakthrough RESEARCH, this process would have benefited from qualitative exploration of the identified domains to aid in the development of the original survey items in phase 1.^22^ This additional step was not feasible due to funding and operational constraints. Second, phase 2 of this process was conducted during the COVID-19 pandemic and was embedded in a large study with multiple data collection activities and supervised remotely, which made data quality assurance more challenging. A consequence of this is that data analysis of phase 2 data was limited to 17 of 20 interviews. Third, phase 3—also embedded as a part of a larger study—had an HCP sample size smaller than the rule of thumb, which is a minimum of 10 participants for each item on the scale.^41^ Despite the small sample size, phase 3 data analyses resulted in robust results. Fourth, the phase 2 sample reflected a different sociodemographic range, in terms of cadre and gender, than that used in phase 3. Best practices dictate that sample characteristics should be kept consistent during scale development. Lastly, we note that this scale development process was conducted in 2 different countries, although both were in francophone sub-Saharan Africa. Cultural context is important in scale development work, where scales are usually developed, validated, and tested in a single country. Thus, our approach, which was limited by operational constraints to continue work in DRC, may have masked cultural or structural differences related to HCPs' perceptions of their professional roles, for example. However, this scale establishes a foundation, and we note the need to continue testing these survey items in different contexts.

## CONCLUSION

Provider attitudes are increasingly recognized as a driver of provider behavior. Service delivery and social behavior change researchers and implementers have identified the need to better understand what provider attitudes might affect health care provision and worker performance. Despite this growing recognition, standardized measurements of provider attitudes, particularly those measuring constructs that could be relevant to multiple health areas, have not been previously developed. Measuring provider attitudes using validated scales such as the scale presented in this article can identify areas for programmatic improvement by helping stakeholders understand drivers of provider behavior. This research highlights not only the result of a measurement development process but also the importance of iteration and testing with the target population in scale development, including the value of qualitative data, such as cognitive interviews, to improve quantitative measures.

Future use and testing of the provider attitude survey items should include considerations for cadre-specific nuances in the understanding of provider attitudes since health providers are not a homogenous group. This would include testing the present scale with a sample that is stratified and powered to detect difference between HCP cadres and gender. Measuring and understanding these differences would help program implementers tailor interventions to shift provider attitudes toward a less authoritarian paradigm. Additionally, this scale was specifically developed to be health-area agnostic, capturing constructs that could relate to provider behaviors across different health areas. Further testing is needed to explore how this scale performs in other contexts and in relation to a range of health outcomes. Service delivery and provider behavior change programs could consider conducting a small number of cognitive interviews to assess whether the constructs captured in the scale items resonate in each local context. Provider behavior change programmers who seek to increase access to and uptake of health services should also address provider attitudes about their professional roles, their clients, and gender norms and further investigate how overall provider attitudes that are more authoritarian in nature could impact the quality of service delivery and affect clients' service uptake and continuation.

## Supplementary Material

GHSP-D-22-00421-supplement.pdf

## Notes

**Peer Reviewed**

**First published online:** August 17, 2023.
