# Supplementary material for: Results From a Multimethod Exploratory Scale Development Process to Measure Authoritarian Provider Attitudes in Democratic Republic of Congo and Togo
Source: Glob Health Sci Pract. 2023 Nov 30;11(Suppl 1):e2200421. doi: 10.9745/GHSP-D-22-00421 (PMC10698232; doi:10.9745/GHSP-D-22-00421)
Supplement: GHSP-D-22-00421-supplement.pdf [file GHSP-D-22-00421-supplement.pdf]

Supplement to: Silva M, Spielman K, Dougherty L, Kalamar A. Measuring authoritarian provider attitudes: a multi-method exploratory scale development process in Democratic Republic of Congo and Togo. *Glob Health Sci Pract*. 2023;11(Suppl 3):e2200421. https://doi.org/10.9745/GHSP-D-22-00421

SUPPLEMENT

TABLE S1. Provider Attitude Survey Items – Phase 1 Descriptive Results

		Strongly Agree	Agree	Neutral	Disagree	Strongly Disagree
	**Attitudes related to perceptions of patients**					
1	Patients I care for are not educated enough make good health decisions for themselves	21.6%	36.0%	12.2%	28.6%	1.7%
2	Patients I care for are not grateful for the efforts I make when I care for them	14.3%	25.1%	11.3%	44.4%	4.9%
3	I consider my patients to be worthy of respect no matter how poor or low status they are	64.4%	33.5%	0.3%	1.4%	0.4%
4	Patients often treat me without respect, so it's hard to treat them with respect	2.1%	3.9%	2.5%	75.4%	16.1%
5	Patients I care for make bad decisions regarding their health no matter what I tell them	6.8%	22.5%	10.8%	55.7%	4.2%
6	My patients will work hard to improve their health when they are given the proper information	38.5%	50.7%	4.9%	5.5%	0.4%
	**Attitudes related to professional role**					
7	My role is to provide clinical care, not to teach patients about how to take care of themselves	7.4%	11.3%	1.7%	64.4%	15.3%
8	I do not spend a lot of thought about what Patients may think about their experience at the clinic as I have other things to worry about	4.8%	5.9%	5.5%	72.2%	11.6%
9	An important part of my job is to communicate with patients to make sure they understand their care	51.5%	44.0%	1.4%	2.4%	0.8%
10	I try hard to think about all of the patients' health care needs not just solving their immediate problem	34.4%	51.1%	3.0%	10.5%	1.0%

		Strongly Agree	Agree	Neutral	Disagree	Strongly Disagree
11	I was trained to provide clinical care, being respectful to every patient is not my job	8.1%	15.7%	3.5%	59.4%	13.3%
12	When medicine is given, it is important that I explain well what it does for the patient and how it helps them	57.7%	39.4%	1.5%	1.1%	0.3%
13	I think it is important to spend enough time with each patient, even if I have other job demands	43.7%	41.9%	6.4%	7.4%	0.6%
14	My job is to diagnose and treat parents not to be a health educator	2.3%	5.4%	2.2%	68.4%	21.6%
15	Engaging patients in discussions leads to better health outcomes than just telling them what is best for them	42.3%	47.1%	3.3%	6.6%	0.7%
	**Gender norms**					
16	A man should have the final word about decisions in his home	35.4%	35.2%	4.4%	21.3%	3.7%
17	It is the man who takes the initiative to have sex with his wife	10.3%	16.5%	12.3%	52.9%	8.1%
18	A women most important role is to take care of her home and cook for her family	14.8%	24.0%	6.6%	47.0%	7.6%
19	If a woman has a good idea, her husband should listen even if he disagrees	46.8%	46.8%	2.7%	3.3%	0.4%
20	Men and women should decide together about how many children to have	58.7%	36.7%	1.5%	2.4%	0.6%
21	A man is expected to discipline his women	18.7%	38.8%	8.5%	27.4%	6.7%
22	Men should help take care of the children in the household	43.5%	48.8%	2.4%	5.0%	0.4%
23	There is never a good reason for a man to beat his wife	34.0%	28.2%	8.6%	25.3%	3.9%

Supplement to: Silva M, Spielman K, Dougherty L, Kalamar A. Measuring authoritarian provider attitudes: a multi-method exploratory scale development process in Democratic Republic of Congo and Togo. *Glob Health Sci Pract*. 2023;11(Suppl 3):e2200421. https://doi.org/10.9745/GHSP-D-22-00421

TABLE S2. French Version of Final Survey Items Retained in Validated Scale

**Scale Items**
Mes patients dont je m'occupe ne sont pas assez capables pour prendre de bonnes décisions pour leur santé.
Mes patients doivent apprécier des efforts que je leur fournis quand je m’occupe d’eux
Il faut traiter les patients avec respect même s’ils ne me traitent pas avec respect
Les patients doivent toujours respecter les prestataires, quelle que soit la qualité des soins qu’ils prodiguent
Mes patients fourniront beaucoup d’efforts pour améliorer leur santé si on leur donne les bonnes informations.
Le rôle d’un prestataire est de diagnostiquer les patients et leur fournir des soins cliniques, et non pas d’apprendre aux patients comment améliorer leur santé et prévenir les maladies
J'ai la responsabilité de veiller à ce que les patients aient leur mot à dire sur les soins qu'ils reçoivent.
Il est important d'écouter les patients pour s'assurer qu'ils comprennent les soins dont ils ont besoin
Mon rôle en tant que prestataire est de résoudre les problèmes médicaux immédiats de mes patients seulement, sans m’occuper d’autre chose
Lorsque des médicaments sont administrés, il est important que j’explique bien aux patients comment ils agissent et en quoi cela va leur être bénéfique.
Mon travail consiste à diagnostiquer et à traiter les patients, pas à être un éducateur de santé pour chaque patient.
Un homme doit avoir le dernier mot sur les décisions prises chez lui
Une femme doit obéir à son mari par rapport à tout
Il est important qu'un père soit présent dans la vie de ses enfants, même s'il n'est plus avec la mère.
